# Economic evaluations in occupational health: what brings the best bang for the buck?

**DOI:** 10.5271/sjweh.4026

**Published:** 2022-04-29

**Authors:** Johanna M van Dongen, Allard J van der Beek

**Affiliations:** 1 Department of Health Sciences, Faculty of Science, Vrije Universiteit Amsterdam, Amsterdam Movement Sciences Research Institute, The Netherlands; 2 Department of Health Sciences, Faculty of Science, Vrije Universiteit Amsterdam, Amsterdam Public Health Research Institute, The Netherlands; 3 Department of Public and Occupational Health, Amsterdam UMC, Vrije Universiteit Amsterdam, Amsterdam Public Health Research Institute, The Netherlands

Worldwide, work-related injury and disease form a major challenge to employers, workers’ unions, governments, and most importantly workers themselves. To combat this, a broad range of occupational health interventions has been developed that are typically aimed at preventing work-related illnesses and disease, and/or improving return-to-work after sickness absence or work disability (1). As resources are restricted, however, decision-makers increasingly call upon advisors and researchers to demonstrate that occupational health interventions are not only effective but also efficient in terms of their resource implications (2). For instance, a qualitative assessment among decision-makers in the Ontario healthcare sector showed that financial information – mostly in the form of a business case – can be a key deciding factor of whether to go forward with implementation of an intervention or occupational health service (3).

In the past decades, various economic studies have been performed in the area of occupational health. These studies can be roughly divided into: (i) studies assessing the cost of ill health among workers and (ii) economic evaluations of interventions (1). Economic evaluations typically assess the cost-effectiveness and/or return-on-investment of occupational health interventions by comparing their incremental costs to their additional effects expressed in terms of physical units and monetary values, respectively. In recent years, various examples of such studies have been published in *The Scandinavian Journal of Work, Environment & Health*. Russo et al (4), for example, reported on the development of a cost-estimation method for work-related stress, while Finnes et al (5) assessed the long-term cost-effectiveness of return-to-work interventions for mental disorder-related absence. In another example, Lutz et al (6) summarized and critically appraised the health economic evidence on worksite physical activity and/or nutrition programs.

Despite these good examples, the methodological quality of many economic evaluations in occupational health is far from optimal (6–8). To illustrate, many economic evaluations used a wide variety of – sometimes invalid – methods for estimating productivity-related costs, few economic evaluations validly assessed and/or reported on the uncertainty surrounding their results, and the follow-up duration of many economic evaluations is relatively short (6–8). Even though various efforts have been made to improve the methodological quality of economic evaluations in occupational health [eg, (2)], three issues still warrant specific attention: (i) measurement and valuation of productivity-related costs, (ii) analysis of trial-based economic evaluations, and (iii) use of modelling techniques. In the following, we will discuss these issues in greater detail and give pointers on where occupational health can benefit from cooperating with other research fields.

**Challenges for economic evaluations in occupational health**

Productivity-related costs are often the central point of focus in both cost of illness studies and economic evaluations in occupation health. This is because the impact of (work-related) injury and disease on productivity-related costs is substantial (9). Hassard et al (10), for example, found that productivity-related costs accounted for 70–90% of the total societal cost of work-related stress. Unfortunately, however, measuring and valuing productivity losses is complex and is likely even further complicated by the broad range of methods available for doing so. To illustrate, sickness absence can be measured objectively using company records or subjectively using a broad range of self-reported questionnaires (e.g. WHO-HPQ, PRODISQ). To complicate matters even further, sickness absence days can be valued using various methodological approaches (eg, the human capital or friction cost approaches) and different kinds of unit prices (eg, actual wage rates, average income) (2, 9). Reduced productivity while being at work (ie, presenteeism), on the other hand, is typically measured using simple numeric rating scales and can again be valued using a wide variety of approaches and unit prices. Consequently, economic evaluations in occupational health are highly heterogeneous in terms of their productivity-related cost estimation methods and large differences exist across studies in terms of the validity and transparent reporting of applied methods (9). We therefore agree with other authors that guidelines should be developed for measuring and valuing productivity-related costs (9). These guidelines should be specific to the field of occupational health and ideally consider cross-country differences. This is important because methods that are appropriate for a certain economic perspective and/or country are not necessarily appropriate for other perspectives and/or countries. In the Netherlands, for example, the “Dutch manual for costing studies in healthcare” recommends to only include productivity losses that occur during the period employers need to replace a sick worker (ie, friction cost approach) (11), whereas – in practice – Dutch employers are obliged to pay ≥70% of their sick workers’ wages for the duration of two years. Hence, it is questionable whether the recommended friction cost approach is appropriate from the company perspective as well. Additionally, we would like to encourage future studies to assess the appropriateness of the simple numeric rating scales that are currently being used for estimating presenteeism costs, and/or whether presenteeism cost estimates are preferably based on more detailed and validated productivity questionnaires, such as the “Individual Work Performance Questionnaire” (12). If the latter is the case, research is needed to assess how to validly convert workers’ responses to such questionnaires into monetary values. Ideally, this is done using objective information on productivity outputs, for instance by systematic worksite observations (13).

The statistical quality of many economic evaluations in occupational health is poor (9, 14). Amongst others, baseline imbalances, the skewed nature of cost data, and the clustering of data are often neglected. On top of that, missing data are frequently handled using inappropriate methods, such as a complete-case analysis, and some studies do not even report on the uncertainty surrounding their cost-effectiveness and/or return-on-investment estimates (8). Inappropriate statistical methods, however, can lead to incorrect results and/or conclusions, and hence a sub-optimal allocation of available occupational health resources (9, 14). Improving the statistical quality of economic evaluations in occupational health starts with the development of recommendations on how to analyze them appropriately, accompanied by the publication of tutorial papers and annotated software codes. Also, as extensive progress has been made concerning the statistical analysis of economic evaluations in other research fields [eg, (14–16)], the quality of economic evaluations in occupational health will also benefit from an increased collaboration with health economists, biostatisticians, and/or econometricians. In the present issue, Finnes et al (5) provide a good example of such a collaboration, which in turn resulted in a state-of-the-art economic evaluation from the occupational health as well as economic perspective.

Most economic evaluations in occupational health are solely based on worker-level data (6–8). Possible limitations of such an approach are that worker-level data are typically only available for a restricted number of workers, relatively short follow-up durations, and a limited number of comparators. To deal with these issues, researchers could make use of analytic modelling where economic evaluations are conducted by synthesizing information from multiple sources (eg, using Markov models) (2). Analytic modelling might, for example, be used to extrapolate economic evaluation results beyond the follow-up of a trial. This may be particularly informative for occupational health interventions that are expected to impact costs and/or effects for an extensive period of time (eg, worksite health promotion programs). Analytic modelling might also be used when researchers want to compare a number of interventions and/or when they are confronted with a large number of uncertain variables. Mofidi et al (17) recently provided a good example of analytic modelling in occupational health by assessing the cost-effectiveness of seven different combinations of silica exposure reduction interventions, while simultaneously assessing the robustness of the study results to a broad range of uncertain factors. Again, as extensive progress concerning analytic modelling has taken place in other research fields [eg, (18)], occupational health researchers aiming to use such techniques will likely benefit from collaborating with other research fields.

**What to do next?**

Economic evaluations in occupational health are confronted with various scientific challenges that need to be addressed to improve their scientific rigor, and hence the validity of their results and conclusions. To tackle some of these challenges, we encourage researchers to develop guidance and recommendations concerning the measurement and valuation of productivity-related costs as well as the statistical analysis of economic evaluations and to increasingly collaborate with researchers from other fields (eg, when aiming to use analytic modelling techniques).

## References

[1] Burdorf A (2007). Economic evaluation in occupational health-its goals, challenges, and opportunities. Scand J Work Environ Health.

[2] van Dongen JM, van Wier MF, Tompa E, Bongers PM, van der Beek AJ, van Tulder MW (2014). Trial-based economic evaluations in occupational health:principles, methods, and recommendations. J Occup Environ Med.

[3] van Dongen JM, Tompa E, Clune L, Sarnocinska-Hart A, Bongers PM, van Tulder MW (2013). Bridging the gap between the economic evaluation literature and daily practice in occupational health:a qualitative study among decision-makers in the healthcare sector. Implement Sci.

[4] Russo S, Ronchetti M, Di Tecco C, Valenti A, Jain A, Mennini FS (2021). Developing a cost-estimation model for work-related stress:An absence-based estimation using data from two Italian case studies. Scand J Work Environ Health.

[5] Finnes A, Hoch JS, Enebrink P, Dahl J, Ghaderi A, Nager A (2022). Economic evaluation of return-to-work interventions for mental disorder-related sickness absence:two years follow-up of a randomized clinical trial. Scand J Work Environ Health.

[6] Lutz N, Clarys P, Koenig I, Deliens T, Taeymans J, Verhaeghe N (2020). Health economic evaluations of interventions to increase physical activity and decrease sedentary behavior at the workplace:a systematic review. Scand J Work Environ Health.

[7] Grimani A, Bergström G, Casallas MIR, Aboagye E, Jensen I, Lohela-Karlssonx M (2018). Economic evaluation of occupational safety and health interventions from the employer perspective:A systematic review. J Occup Environ Med.

[8] van Dongen JM, Proper KI, van Wier MF, van der Beek AJ, Bongers PM, van Mechelen W (2012). A systematic review of the cost-effectiveness of worksite physical activity and/or nutrition programs. Scand J Work Environ Health.

[9] Steel J, Godderis L, Luyten J (2018). Productivity estimation in economic evaluations of occupational health and safety interventions:a systematic review. Scand J Work Environ Health.

[10] Hassard J, Teoh KR, Visockaite G, Dewe P, Cox T (2018). The cost of work-related stress to society:A systematic review. J Occup Health Psychol.

[11] Versteegh M, Knies S, Brouwer W (2016). From good to better:new Dutch guidelines for economic evaluations in healthcare. Pharmacoeconomics.

[12] Koopmans L, Bernaards C, Hildebrandt V, van Buuren S, van der Beek AJ, dex Vet HC (2013). Development of an individual work performance questionnaire. Int J Product Perform Manag.

[13] Meerding WJ, IJzelenberg W, Koopmanschap MA, Severens JL, Burdorf A (2005). Health problems lead to considerable productivity loss at work among workers with high physical load jobs. J Clin Epidemiol.

[14] Mutubuki EN, El Alili M, Bosmans JE, Oosterhuis T, J Snoek F, Ostelo RWJG (2021). The statistical approach in trial-based economic evaluations matters:get your statistics together!. BMC Health Serv Res.

[15] Gomes M, Grieve R, Nixon R, Edmunds WJ (2012). Statistical methods for cost-effectiveness analyses that use data from cluster randomized trials:a systematic review and checklist for critical appraisal. Med Decis Making.

[16] Faria R, Gomes M, Epstein D, White IR (2014). A guide to handling missing data in cost-effectiveness analysis conducted within randomised controlled trials. Pharmacoeconomics.

[17] Mofidi A, Tompa E, Mortazavi SB, Esfahanipour A, Demers PA (2020). A probabilistic approach for economic evaluation of occupational health and safety interventions:a case study of silica exposure reduction interventions in the construction sector. BMC Public Health.

[18] Krijkamp EM, Alarid-Escudero F, Enns EA, Jalal HJ, Hunink MM, Pechlivanoglou P (2018). Microsimulation modeling for health decision sciences using R:a tutorial. Med Decis Making.

